# Clinical Lectures on Diseases of Women

**Published:** 1863-02

**Authors:** 


CLINICAL LECTURES ON DISEASES OF WOMEN. By J. Y. SIMPSON, M.D., F.R.S.E., Professor of Midwifery in the University of Edinburgh, etc. Illustrated with 102 Engravings on Wood. LEA & BLANCHARD. Price $3. For Sale by W. B. KEEN, Chicago.

This is a collection of lectures originally published in the *London Medical Times and Gazette*, during the years 1859, 1860, and 1861; and first reprinted by LEA & BLANCHARD in *The Medical News and Library*, in the same style that they published in the same journal *West's Lectures on Diseases of Females*, *Todd on Acute Diseases*, and other works. This is a fair octvao of 510 pages, very lightly bound in cloth. The wood-cuts are unequal,—some being very good, and some not worthy of the high character of the work. As a contribution to the pages of a monthly journal, or illustrations of a brochure, (which word seems to be accepted in English as meaning a little

*more* than a "stitched pamphlet,") they were well enough. When transferred to a book of the claims of this one, they might well have been improved; however, in these days of high prices, the book will be acceptable for containing more than the money's worth.

It is needless to say anything to the professional reader of the character of Dr. SIMPSON as an observer in medicine, or as an author. In both respects, he is well known to hold the very front rank in the profession. In this volume, he sustains his former reputation. The style is singularly attractive, while it is wholly devoid of ornament, or anything else than the direct and simple discussion of the topics considered.

It is a singular fact, that in a style of such real excellence and perspicuity too, we should find the term "sedative" used as synonymous with "anodyne."

Professor SIMPSON seems to have felt somewhat forcibly the fact: that many surgeons are disposed to claim that their department of the profession has obtained the highest advancement and perfection, "forgetting that it is the mere handicraft part of it that has been brought to this boasted degree of development." To meet this assumption, he quotes from Dr. GREGORY a rejoinder to that assumption, premising that at the time Dr. GREGORY spoke, there were in Edinburgh two surgeons, rivals, and both authors of large treatises on surgery, named Mr. BENJAMIN BELL and Mr. JOHN BELL:—

"Within my memory, a new mode of cutting off legs was introduced, (or an old one revived,) and strongly recommended by an eminent surgeon, Mr. ALANSON. It was called the flap operation, or cutting with flaps. I remember to have heard some disputes about it; for, as there were flappers, of course there must have been anti-flappers; and as the dispute began little more than twenty years ago, far from being ended as yet, it can scarce be arrived at full maturity and violence. Mr. BENJAMIN BELL must either be a flapper or an anti-flapper; and I humbly conjecture, (for I do not know the fact,) that if he is a flapper, Mr. JOHN BELL will be a determined anti-flapper; but that if BENJAMIN is a anti-flapper, JOHN will be a most strenuous flapper; but flap or no flap, he certainly may take his choice of several ways of cutting off a leg.

Professor SIMPSON proceeds to remark that the controversy yet goes on now, (some thirty years later;) and the surgeons forget to ask themselves: "why it is that so many of their patients die after every possible form and fashion of dismemberment, and how this mortality may possibly be met and diminished?"

We think that this charge will not lie against American surgeons; but there is a tendency in the war to magnify surgery, somewhat at the expense of the study of the Principles of Medicine, upon which, both departments of our profession depend for their highest success. Whilst the war certainly creates a necessity for a large amount of surgery, the art is far secondary, even in the military service, to practical medicine; and, while it is certainly right, that the opening of this somewhat wider field for surgery, should give a new impulse to the study of surgical anatomy and operative surgery, a still greater attention ought to be given to medical science; and we trust that the practical good sense of our army will secure this result.

